# Photoelectrocatalytic Synthesis of Hydrogen Peroxide by Molecular Copper‐Porphyrin Supported on Titanium Dioxide Nanotubes

**DOI:** 10.1002/cctc.201702055

**Published:** 2018-02-20

**Authors:** Dogukan H. Apaydin, Hathaichanok Seelajaroen, Orathip Pengsakul, Patchanita Thamyongkit, Niyazi Serdar Sariciftci, Julia Kunze‐Liebhäuser, Engelbert Portenkirchner

**Affiliations:** ^1^ Linz Institute for Organic Solar Cells (LIOS), Institute of Physical Chemistry Johannes Kepler University Linz 4040 Linz Austria; ^2^ Petrochemistry and Polymer Science Program, Faculty of Science Chulalongkorn University Bangkok 10330 Thailand; ^3^ Department of Chemistry, Faculty of Science Chulalongkorn University Bangkok 10330 Thailand; ^4^ Research group on Materials for Clean Energy Production STAR, Department of Chemistry, Faculty of Science Chulalongkorn University Bangkok 10330 Thailand; ^5^ Institute of Physical Chemistry University of Innsbruck 6020 Innsbruck Austria

**Keywords:** copper, heterogeneous catalysis, hydrogen peroxide, oxygen reduction, photoelectrochemistry

## Abstract

We report on a self‐assembled system comprising a molecular copper‐porphyrin photoelectrocatalyst, 5‐(4‐carboxy‐phenyl)‐10,15,20‐triphenylporphyrinatocopper(II) (CuTPP‐COOH), covalently bound to self‐organized, anodic titania nanotube arrays (TiO_2_ NTs) for photoelectrochemical reduction of oxygen. Visible light irradiation of the porphyrin‐covered TiO_2_ NTs under cathodic polarization up to −0.3 V vs. Normal hydrogen electrode (NHE) photocatalytically produces H_2_O_2_ in pH neutral electrolyte, at room temperature and without need of sacrificial electron donors. The formation of H_2_O_2_ upon irradiation is proven and quantified by direct colorimetric detection using 4‐nitrophenyl boronic acid (*p*‐NPBA) as a reactant. This simple approach for the attachment of a small molecular catalyst to TiO_2_ NTs may ultimately allow for the preparation of a low‐cost H_2_O_2_ evolving cathode for efficient photoelectrochemical energy storage under ambient conditions.

Two‐electron oxygen reduction reaction (ORR) of dissolved oxygen (O_2_) in water leads to formation of hydrogen peroxide (H_2_O_2_) which is a versatile, high energy product,^1^ capable of participating in numerous further redox reactions and is an active species in a plethora of biological processes.^2^ Solar‐driven H_2_O_2_ formation has been proposed for chemical energy storage.^1^, ^3^, ^4^, ^5^ However, the widely used anthraquinone process for the formation of H_2_O_2_ is known to be energy intensive.^6^ For many decades, researchers have tried to address this issue and tackle the problem by introducing metal catalysts,^7^, ^8^, ^9^, ^10^, ^11^, ^12^ core–shell structures, metal oxides, metal chalcogenides etc.^13^, ^14^, ^15^ Additionally, photocatalytic reduction of O_2_ to H_2_O_2_ by inorganic semiconductors (e.g. are ZnO, CdS and TiO_2_) and organometallic complexes has been reported.^14^, ^16^, ^17^, ^18^ Recently, metal‐free carbon‐based catalysts has been the focus for (photo)electrochemical reduction of dissolved O_2_. This class mainly includes graphitic carbon nitrides (g‐C_3_N_4_) and organic pigments.^19^, ^20^, ^21^ However, almost all of these reactions require either acidic or basic conditions which make daily applications challenging. Although there are a few examples,^22^ the search for a catalyst which works under mild pH conditions is still in progress.

Here, we present a photoelectrode consisting of a porphyrin derivative, namely CuTPP‐COOH (Figure 1 a), coated on TiO_2_ nanotubes (NT) (TiO_2_ NTs/CuTPP‐COOH) for the reduction of O_2_ to H_2_O_2_. The introduction of a carboxyl group enables the attachment of the photoactive porphyrin onto the nanostructured TiO_2_ NTs.^23^ CuTPP‐COOH was chosen owing to ease of its synthesis as well as the appropriate energy levels to reduce O_2_. We have also utilized ZnTPP‐COOH for the same reaction; however the stability of this material was inferior. The high surface area of TiO_2_ NTs^24^ increases the number of potential catalytically active sites . In addition, the amorphous structure of TiO_2_ NTs helps to anchor the CuTPP‐COOH through the −COOH functional group. The reaction takes place at neutral pH and ambient temperature (22 °C). By applying moderate negative potentials between 0.0 V and −0.3 V vs. NHE (normal hydrogen electrode) and upon photoexcitation of CuTPP‐COOH at *λ*>395 nm, an exciton (electron‐hole pair) is initially formed, as illustrated in Figure 1 b. Subsequently, the newly created hole residing in the valence band of CuTPP‐COOH is recombined with an electron supplied from the external circuit, while the electron in the conduction band is capable of reducing the dissolved O_2_ in water to H_2_O_2_.

**Figure 1:**
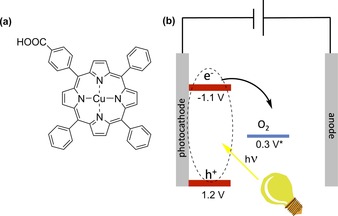
(a) Chemical structure of CuTPP‐COOH, and (b) schematic representation of photoelectrochemical reduction of O_2_. Formal potential of O_2_ reduction to H_2_O_2_ is recalculated for pH7 from the reported literature values.^25^

Characterization of the TiO_2_ NTs/CuTPP‐COOH films using scanning electron micrographs (SEM, Figure S2, Supporting Information), optical imaging (Figure S3, Supporting Information) and Fourier‐transform infrared spectroscopy (FTIR, Figure S4, Supporting Information) techniques is presented in the Supporting Information.

The electrochemical behavior of the TiO_2_ NTs/CuTPP‐COOH photoelectrodes under applied potential in Ar‐ and O_2_‐saturated conditions can be seen in Figure 2. In the absence of O_2_, the illumination led to no observable increase in current with a current density (*j*) of approximately 1.3 μA cm^−2^ at −0.3 V vs. NHE. However, under O_2_ saturation and upon light illumination, the current value increased around 4 fold and reached approximately 13 μA cm^−2^ at −0.3 V, signaling the reduction of dissolved O_2_.

**Figure 2:**
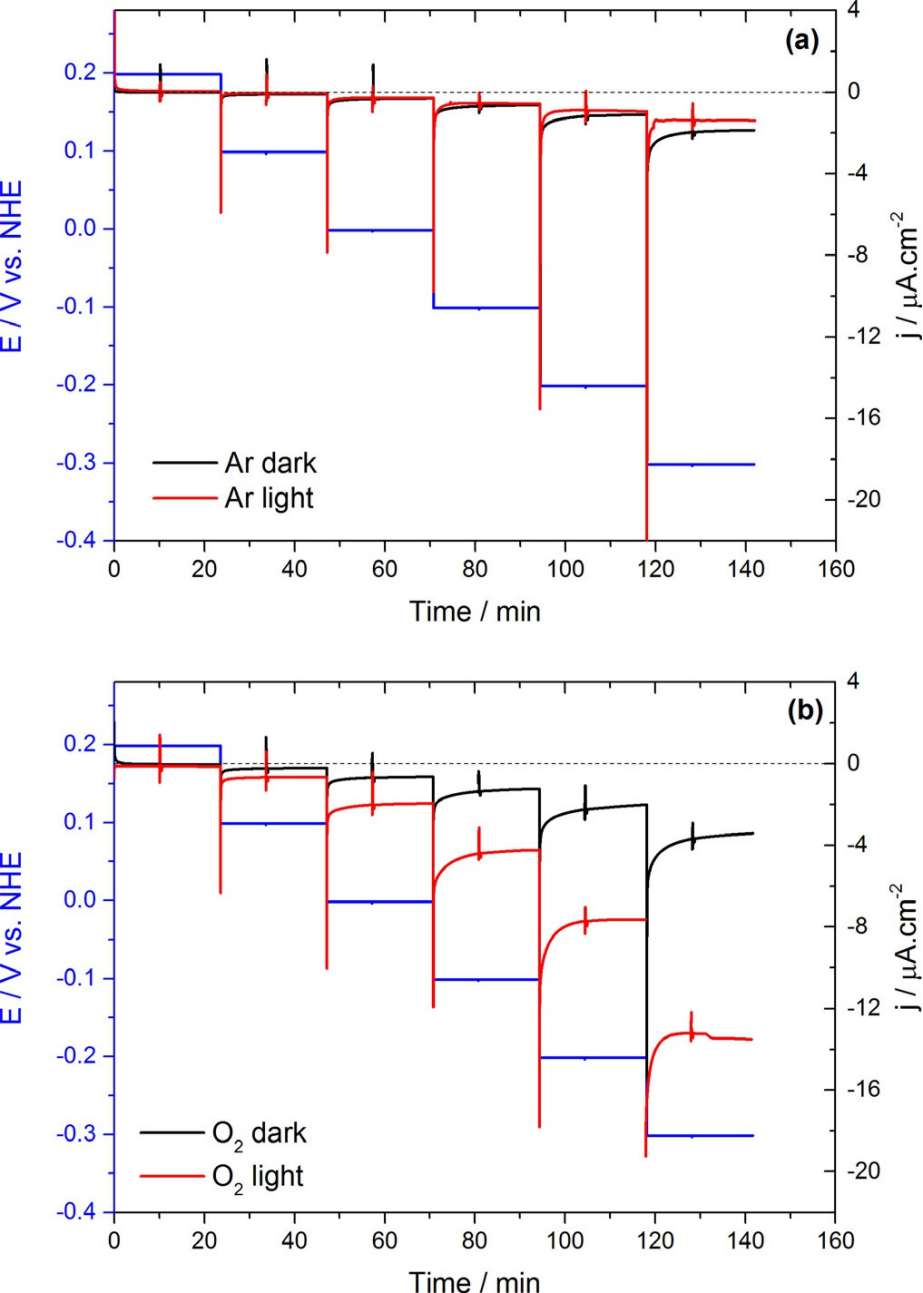
Chronoamperometry of CuTPP‐COOH‐coated electrodes in the dark (black solid line) and upon illumination (red solid line) under (a) Ar and (b) under O_2_ saturation. An aqueous solution of 0.1 m Na_2_SO_4_ was used as the electrolyte. In both graphs the blue solid line shows the applied potential.

After chronoamperometry experiments, a series of constant potential electrolysis experiments were conducted to quantify the formation of H_2_O_2_. One of the reasonable ways for direct detection of H_2_O_2_, is an indirect spectrophotometric method for the quantification of the product, relying on a stoichiometric reaction of arylboronic acids with newly generated H_2_O_2_ under mild basic conditions to yield the respective photoactive phenolates.^26^, ^27^ In this work, *p*‐nitrophenylboronic acid (*p*‐NPBA) was used at pH 9, which was converted upon reaction with H_2_O_2_ into *p*‐nitrophenol (*p*‐NP), for which absorption could be observed by using UV/Vis spectrophotometry at 405 nm. A calibration curve for quantitative determination of H_2_O_2_ in a concentration range between 0.5 μm and 20 μm is shown in Figure 3. The detailed procedure for the preparation of the standard solutions can be found in the Supporting Information.

**Figure 3:**
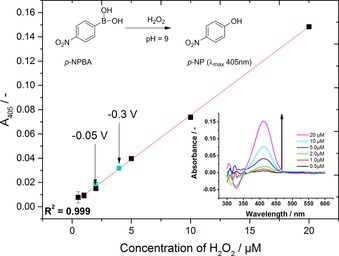
Calibration curve used for quantifying the produced H_2_O_2_. Reaction leading to *p*‐NP formation (upper left inset). Increase in absorbance with increasing concentration of H_2_O_2_ (lower right inset). Points with turquoise color are the concentrations of H_2_O_2_ obtained from electrolysis at constant potentials of −0.05 V and −0.3 V vs. NHE.

After each constant potential electrolysis measurement, an aliquot of 100 μL was pipetted from the electrolyte solution and then transferred into a vial containing the *p*‐NPBA and carbonate buffer. Amounts of H_2_O_2_, reflected by those of newly formed *p*‐NP, between 1.9 μm and 3.9 μm were observed at −0.05 V and −0.3 V applied bias, respectively. The Figure of merit for comparing different H_2_O_2_ forming catalysts is a formation rate which is given in μg_H2O2_ mg_cat_^‐1^ h^‐1^. Nanostructured TiO_2_ supported CuTPP‐COOH electrodes reached the formation rates of 2.2 μg_H2O2_ mg_CuTPP‐COOH_^‐1^ h^‐1^ and 13.4 μg_H2O2_ mg_CuTPP‐COOH_^‐1^ h^‐1^ for the applied potentials of −0.05 V and −0.3 V, respectively. Our system is comparable to well‐known semiconductors such as ZnO (21 μg mg_cat_^−1^ h^−1^)^28^ and g‐C_3_N_4_ (4.25 μg mg_cat_^−1^ h^−1^)^19^, ^29^ Corresponding control experiments in which the O_2_‐saturated solution was measured in the dark did not yield any detectable amount of H_2_O_2_.

To further evaluate the electrochemical characteristics of the O_2_ reduction on the TiO_2_ NTs**/**CuTPP‐COOH photoelectrodes, we conducted potential‐dependent electrochemical impedance spectroscopy (PEIS, Figure 4). Symbols represent experimental data and lines the best fits. The spectra were collected in the potential range between 0.2 and −0.3 V with a step size of 0.1 V. Each potential was kept constant for 10 min to ensure steady state conditions before the impedance measurement, ranging from 100 kHz to 20 mHz, with a peak amplitude of ±10 mV. Measurements were performed under illumination in the Ar‐ and O_2_‐saturated electrolyte solution containing 0.1 m Na_2_SO_4_.

**Figure 4:**
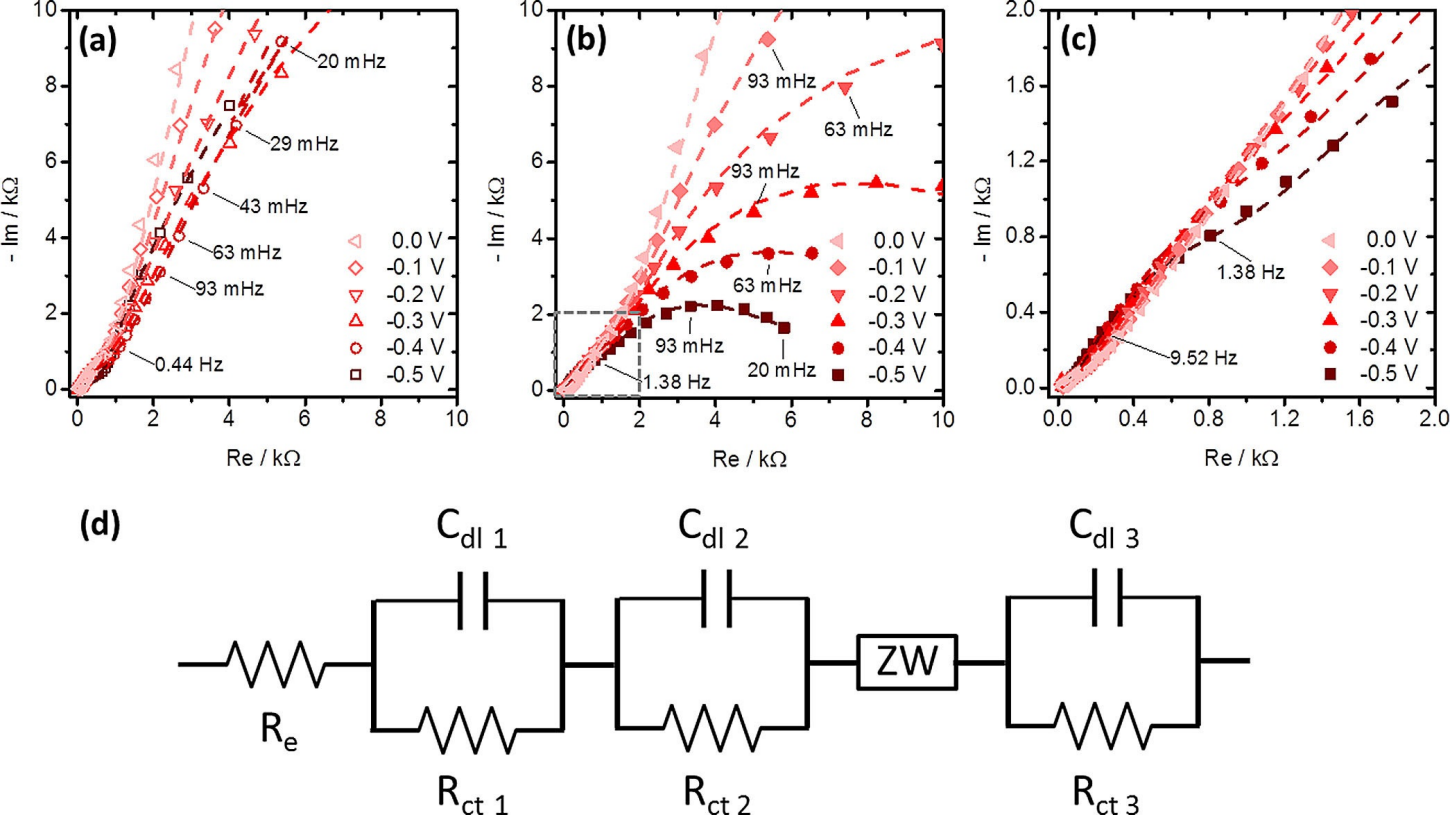
Nyquist plots at different potentials for illuminated, porphyrin covered TiO_2_ NTs under (a) Ar and (b) O_2_ saturation in the 0.1 m Na_2_SO_4_ solution. Symbols represent the experimental data and the lines of best fit. (c) Enlarged view of the high frequency domain of (b) indicated therein with a grey, dashed square. (d) Equivalent electric circuit used for fitting the EIS data. *R*_s_: solution resistance, *R*_f_ and *C*_f_: interfacial TiO_2_/CuTPP‐COOH electron charge transfer resistance and the corresponding capacitance, *R*_tr_ and CPE_nt_: resistance for electron transport along the TiO_2_ NTs and the corresponding capacitance (modelled with a CPE), *Z*W: Warburg element for semi‐infinite diffusion, *R*_r_ and CPE_r_: charge transfer resistance for the O_2_ reduction and corresponding capacitance (modelled with a CPE).

Detailed analysis of Nyquist plots under O_2_ saturation reveals the presence of three, not fully developed semi circles (Figure S5, Supporting Information) The first semi‐circle (I) at high frequencies between 4.5 kHz and 200 Hz was observable in all spectra and may describe the interfacial TiO_2_/CuTPP‐COOH charge transfer. A second semi‐circle (II) at medium frequencies between 65 Hz and 1.4 Hz is also observable in all spectra and may represent the resistance for electron transport along the TiO_2_ NTs and the corresponding surface capacitance.^30^ The development of an additional semi‐circle (III) at potentials below 0.0 V and lower frequencies between 0.94 Hz and 20 mHz is observable only if the electrolyte is saturated with O_2_. This may correspond to the charge transfer resistance of the O_2_ reduction reaction. A two‐step reaction process is expected to be the reason for the occurrence of semi‐circle (III), for example an intermediate state that is involved.^31^

For further quantification of the measured EIS data, corresponding electronic elements were determined by fitting the experimental spectra to the proposed equivalent circuit depicted in Figure 4 d. The proposed equivalent circuit is a modified version of the equivalent circuit introduces by Köleli et.al. for CO_2_ reduction on polyaniline‐coated electrodes.^31^

An additional R/C element at high frequencies has been added to account for the TiO_2_/CuTPP‐COOH interface at the nanostructured support‐electrodes, partly adopted from the transmission line model originally introduced for nanostructured TiO_2_ hybrid solar cells.^30^ The real capacitors C_nt_ and C_r_ are modeled with CPEs to account for the non‐ideal behavior (i.e. depressed semi‐circle) of the capacitive part at medium and low frequencies.^32^ The finite length Warburg impedance (ZW) is used to describe the transport phenomena of O_2_ into the porphyrin film and the transport of reduction products out of the film. The parallel configuration of the *R*_tr_/CPE_nt_ and *R*_r_/CPE_r_ elements may be justified owing to the inhomogeneity (porosity) of the CuTPP‐COOH covered TiO_2_ NTs. From the EIS data we concluded that the ohmic resistance of the electrolyte solution (*R*_s_) is almost constant at all potentials, fluctuating slightly between 17 and 20 Ω. The *R*_f_ (interfacial electron charge transfer resistance) is relatively high at positive potentials with 111.6 kΩ at 0.2 V and decreases significantly to 3.1 kΩ at −0.3 V. This suggests enhanced charge transfer over the TiO_2_/CuTPP‐COOH interface with applied negative bias. *R*_tr_, which describes the resistance for electron transport along the TiO_2_ NTs, decreases only slightly with the applied potential from initially 2.4 kΩ at 0.2 V to 158 Ω at −0.3 V. This characteristic behavior of *R*_tr_ is expected for relatively highly doped nanotubes suggesting a small variation of the carrier density with bias (unless full depletion is obtained).^30^ The charge transfer resistance related to the O_2_ reduction reaction (*R*_r_) could not be determined for positive potentials of 0.2 and 0.1 V, respectively, since the corresponding semi‐circle was not developed in the measured frequency limit (20 mHz). Therefore, it was sufficient to fit the electrochemical impedance spectroscopy data at 0.2 V and 0.1 V without the electronic elements used for describing the O_2_ diffusion and reduction reaction (ZW, *R*_r_ and CPE_r_). At 0.0 V the occurrence of semi‐circle (III) becomes notable and *R*_r_ was determined with 214 kΩ. *R*_r_ then decreased significantly to about 2.3 kΩ at −0.3 V, suggesting enhanced O_2_ reduction at lower potentials. This is congruent with the observed characteristics from chronoamperometry experiments (Figure 2). Overall, the authors are fully aware that the proposed equivalent circuit may not cope with the complexity of the investigated system and was introduced only as an initial attempt to describe the measured EIS data. Also one has to point out that the EIS measurements were not performed under diffusion controlled conditions (i.e. by a rotating disk electrode), rendering its interpretation challenging. Nonetheless, the proposed equivalent circuit demonstrated good fitting congruency in the Nyquist and Bode plots (Figure 4 b and Figure S6, Supporting Information), with, for example, a mean square deviation (X^2^/Z) of 0.3 % for the EIS data recorded at −0.3 V under O_2_ saturation (Figure 4 b). A detailed summary of all fitting parameters and their corresponding mean square deviations is given in Table S1, and a comparison of all impedance measurements under Ar and O_2_ saturation is shown in Figure S7, in the Supporting Information.

In summary, we have demonstrated a novel photocathode capable of reducing dissolved O_2_ to H_2_O_2_ with evolution rates ranging between 2 and 13 μg_H2O2_ mg_cat_^‐1^ h^‐1^. By attaching the CuTPP‐COOH catalyst onto TiO_2_ NTs through its carboxyl group, we created a heterogeneous molecular catalyst where the formed photocathode is convenient for use in aqueous medium and inherits a significantly higher surface area over planar electrodes owing to self‐organized nanostructured TiO_2_ NTs. The TiO_2_ nanostructures were previously used as catalytic moieties together with sensitizers such as porphyrins and phthalocyanines. However, the use of such molecular porphyrins as photoelectrocatalysts is not common. We have also demonstrated that our system is capable of driving the aforementioned reaction under pH neutral conditions which is expected to reduce the technical complications originating from high acidic or alkaline media.

## Experimental Section

Experimental Details such as the synthesis and the details of the electrochemical setup as well as electrochemical impedance spectroscopy can be found in Supporting Information.

## Conflict of interest

*The authors declare no conflict of interest*.

## Supporting information

As a service to our authors and readers, this journal provides supporting information supplied by the authors. Such materials are peer reviewed and may be re‐organized for online delivery, but are not copy‐edited or typeset. Technical support issues arising from supporting information (other than missing files) should be addressed to the authors.

Supplementary
