# Supplementary material for: Photoelectrocatalytic Synthesis of Hydrogen Peroxide by Molecular Copper‐Porphyrin Supported on Titanium Dioxide Nanotubes
Source: ChemCatChem. 2018 Feb 20;10(8):1793–7. doi: 10.1002/cctc.201702055 (PMC5947148; doi:10.1002/cctc.201702055)
Supplement: Supplementary file 1 — Supplementary [file CCTC-10-1793-s001.pdf]

Heterogeneous & Homogeneous & Bio- & Nano-
CHEMCATCHEM
CATALYSIS

Supporting Information

Photoelectrocatalytic Synthesis of Hydrogen Peroxide by Molecular Copper-Porphyrin Supported on Titanium Dioxide Nanotubes

Dogukan H. Apaydin,*[a] Hathaichanok Seelajaroen,[a] Orathip Pengsakul,[b] Patchanita Thamyongkit,[c, d] Niyazi Serdar Sariciftci,[a] Julia Kunze-Liebhäuser,[e] and Engelbert Portenkirchner*[e]

cctc_201702055_sm_miscellaneous_information.pdf

S1. Synthesis of 5-(4-carboxyphenyl)-10,15,20-triphenylporphyrinatocopper(II) (CuTPP-COOH)

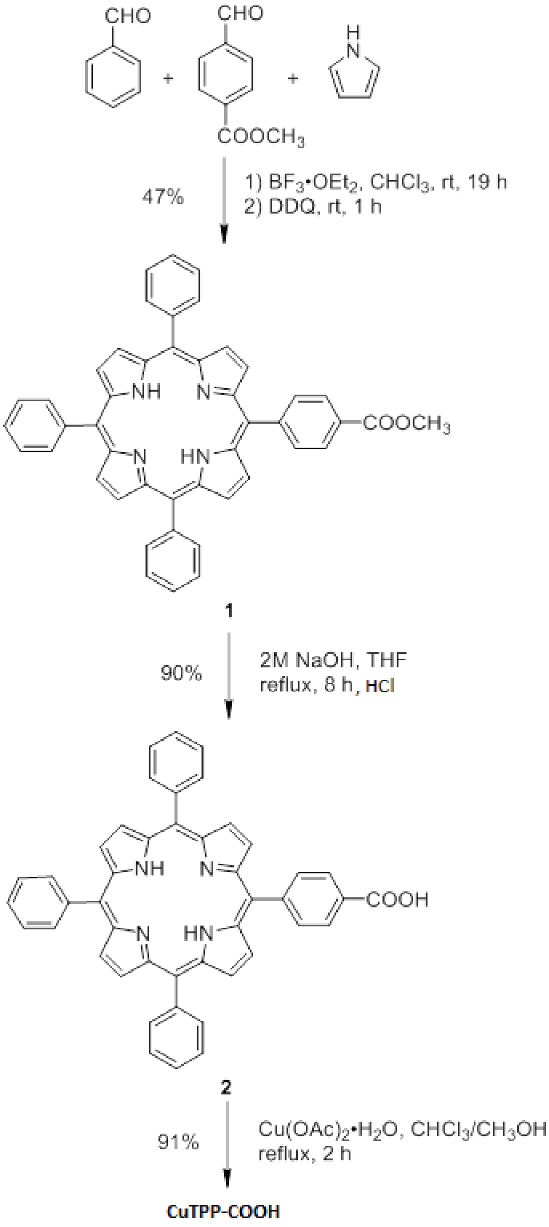

Scheme S1: Synthesis of **CuTPP-COOH**

Materials and methods

^{1}H-NMR (400 MHz) and ^{13}C-NMR (100 MHz) spectra were recorded in $CDCl_3$. Chemical shifts (δ) are reported in parts per million (ppm) relative to the residual $CHCl_3$ peak 7.26 ppm for ^{1}H-NMR and 77.0 ppm for ^{13}C-NMR spectroscopy. Coupling constant (*J*) are reported in Hertz (Hz). Mass spectra were obtained using matrix-assisted laser desorption ionization (MALDI) mass spectrometry using dithranol as a matrix. Absorption spectra were recorded

in toluene at room temperature by a Hewlett-Packard 8453 spectrophotometer and absorption extinction coefficient (ε) were reported in $L \cdot mol^{-1} \cdot cm^{-1}$.

5-(4-Carboxyphenyl)-10,15,20-triphenylporphyrinatocopper(II) (CuTPP-COOH)

With a slight modification of a previous procedure,[1] a mixture of pyrrole (1.55 mL, 22.3 mmol), benzaldehyde (1.85 mL, 18.5 mmol) and methyl 4-formylbenzoate (1.000 g, 6.150 mmol) in $CHCl_3$ (400 mL) was treated with $BF_3 \cdot OEt_2$ (0.01 mL, 0.13 mmol) at room temperature for 19 h. After that, 2,3-dichloro-5,6-dicyano-1,4-benzoquinone (DDQ) (2.550 g, 11.15 mmol) was added and stirred at room temperature for an additional hour. To quench the reaction, triethylamine (1 mL) was added. After removal of the solvent, the resulting crude product was purified by a silica column (CH_2Cl_2/hexanes = 4:1) to afford a compound **1** as a purple solid (1.739 g, 47%). ^{1}H-NMR ($CDCl_3$) δ −2.86 (s, 2H), 4.04 (s, 3H), 7.69 (q, *J* = 6.8 Hz, 4H), 8.12−8.17 (m, 8H), 8.30 (dd, *J* = 8.4, 8.4 Hz, 8H), 8.77 (s, 8H); MALDI-TOF-MS *m/z* (%): found, 671.846 [M^+] calcd, 672.253 (M^+, M = $C_{46}H_{32}N_4O_2$). Other spectroscopic data are consistent with those described in the literature.

In the next step, a solution of **1** (0.100 g, 0.149 mmol) in tetrahydrofuran (THF) (5 mL) was refluxed with a 2M aqueous NaOH solution (10 mL) for 8 h. After cooling down the reaction mixture to room temperature, the resulting solution was neutralized with diluted HCl and extracted with ethyl acetate. The organic mixture was dried over anhydrous Na_2SO_4, filtered and concentrated to dryness. After removal of the solvents, the crude was purified by a silica column using 5% methanol in CH_2Cl_2 to afford a purple solid as a compound **2** (0.089 g, 90%). ^{1}H-NMR ($CDCl_3$) δ −2.78 (s, 2H), 7.76−7.78 (m, 8H), 8.22 (d, *J* = 5.2 Hz, 8H), 8.44 (dd, *J* = 8.4, 7.2 Hz, 8H); ^{13}C-NMR ($CDCl_3$) δ 118.8, 120.3, 126.5, 127.6, 129.4, 130.5, 132.2, 134.2, 142.2, 146.5, 166.8; MALDI-TOF-MS *m/z* (%): found 658.985 [M^+]; calcd 658.745 (M^+, M=$C_{45}H_{30}N_4O_2$); λ_{abs} (ε) 416 (5.7×10^5), 513, 570, 626 nm. Due to low absorption of Q-bands, ε at those wavelengths could not be determined.

With a slight modification from a previous procedure,[2] a solution of **2** (0.200 g, 0.202 mmol) in $CHCl_3$ (80 mL) was refluxed with a solution of $Cu(OAc)_2 \cdot H_2O$ (0.200 g, 1.00 mmol) in methanol (20 mL) for 2 h. After removal of the solvents, the resulting crude was purified by a silica column using 5% methanol in CH_2Cl_2 to afford a red-purple solid as a compound **Cu-2** (0.132 g, 91%). MALDI-TOF-MS *m/z* (%): found 718.768 [M^+], calcd 720.275 (M^+, M = $C_{45}H_{28}N_4O_2Cu$). λ_{abs} (ε) 415 (4.2×10^5), 540, 633 nm (**Figure S1).** Due to very low absorbance of Q-bands, ε at those wavelengths could not be determined.

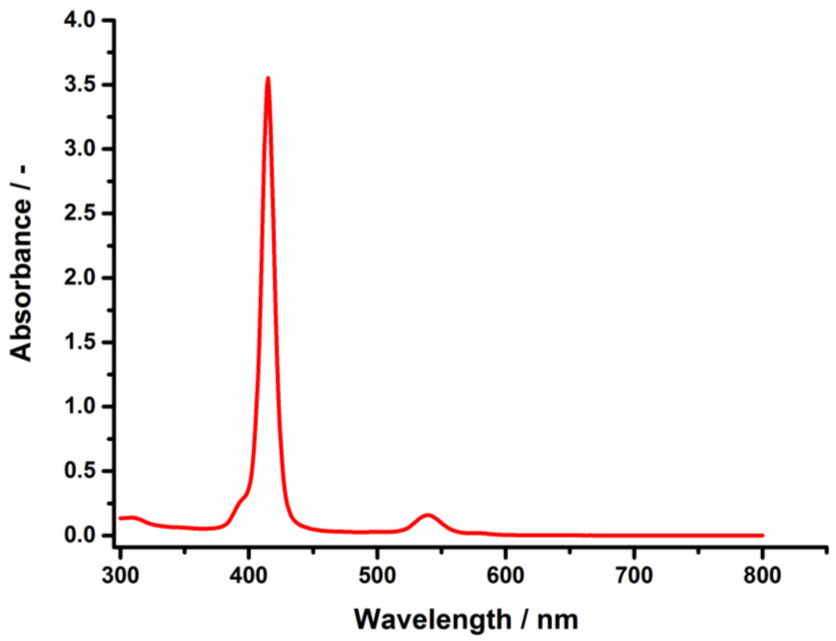

Figure S1. UV-Vis spectrum of **CuTPP-COOH**

S2. Preparation and Characterization of TiO_2 Nanotubes

Nanotube synthesis: TiO_2 NTs were grown electrochemically on mechanically polished (4000P SiC grinding paper) Ti disks (99.6 %, Advent) by exposing the polished surface (2.54 cm^2) to an electrolyte containing 50 vol% ethylene glycol (99.5 %, Merck) in water (18.2 MΩ cm, Milli-Q, Millipore) and 1 wt. % NH_4F (99.99 %, Merck) and by applying an anodic potential of 20 V for 1 h, after a voltage ramp of 1 V s^{-1}.

We have characterized the TiO_2 NTs coated with CuTPP-COOH films using SEM (**Figure S2**), optical imaging (**Figure S3**) and FTIR (**Figure S4**) techniques.

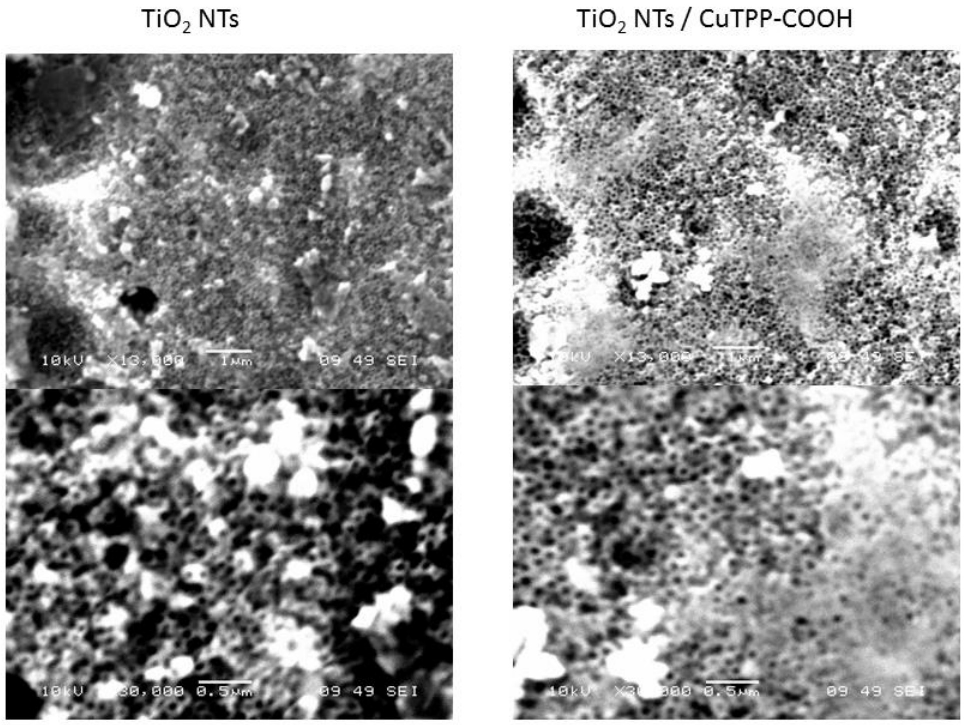

Figure S2. SEM images of TiO_2 NTs (on the left) and TiO_2 NTs coated with CuTPP-COOH (on the right).

As it can be seen from the SEM images after coating of the surface with CuTPP-COOH there is a thin film appearing over the tubes indicating the presence of CuTPP-COOH on the surface. In addition we have made the photos of the electrodes before and after coating (**Figure S3**).

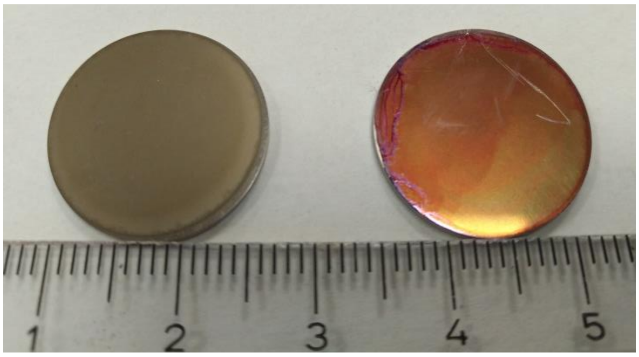

Figure S3. Photo of TiO_2 Nts (on the left) and TiO_2 NTs coated with CuTPP-COOH (on the right).

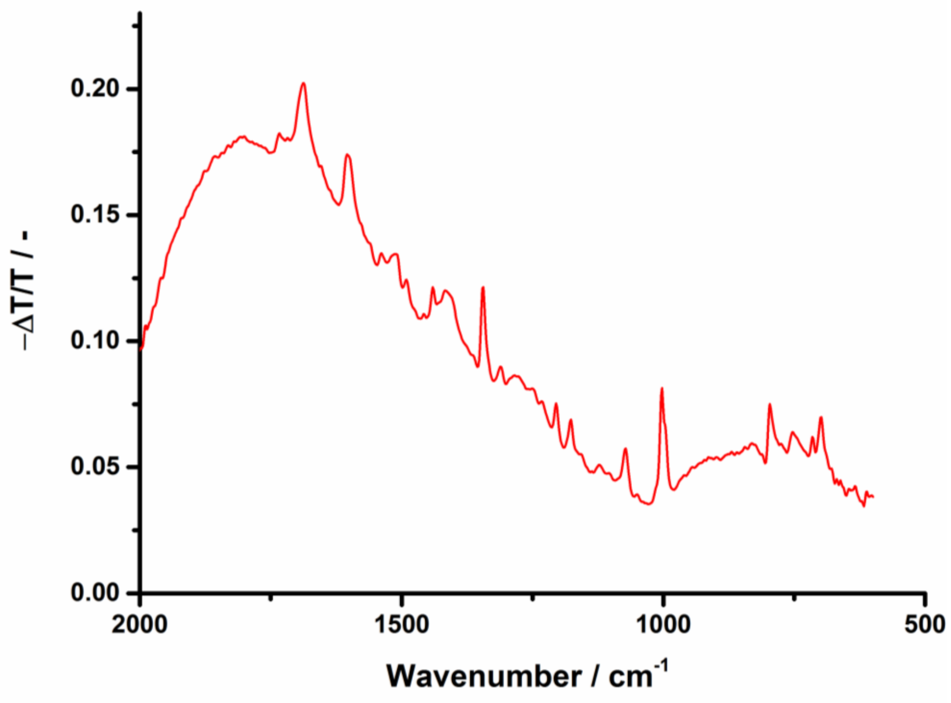

Figure S4. FTIR spectra of the thin films in attenuated total reflection (ATR) mode by pressing the electrodes onto a diamond reflection element.

-ΔT/T corresponds to the absorption calculated from the ATR mode of FTIR. For this first a TiO_2 NTs bare electrode is measured and then the electrode is coated with CuTPP-COOH. This newly assembled electrode is also measured in ATR mode and the above spectrum is calculated eliminating all the features coming from the bare electrode. In this case the peaks pointing upwards correspond to the evolving features and the peaks pointing downwards correspond to the peaks disappearing. The peak around 1685 cm^{-1} corresponds to the ketone formed by anchoring of –COOH to the surface oxide. The peak at 1342 cm^{-1} is indicating the aromatic C-N stretching mode.

S3. Electrochemical Experiments

All electrochemical experiments were done in a one-compartment cell consisting of three electrodes. Nanostructured TiO_2 covered with **CuTPP-COOH** served as the working electrode. In order to avoid side reactions which might originate from TiO_2 itself, the electrode is covered with a chemical resistant Teflon tape allowing a 1.54 cm^2 area exposed to the electrolyte. An Ag/AgCl (3M KCl) electrode served as the reference electrode and a Pt plate served as counter electrode. A long pass filter (λ = 395nm) was used in order to avoid UV excitation of TiO_2 substrate. A 0.1M Na_2SO_4 solution in 18 MΩ water was used as the

electrolyte solution. Prior to any electrochemical experiments the electrolyte solution was either purged with Ar or O_2 to saturation. Electrochemical impedance spectroscopy (EIS) measurements were recorded using a BioLogic VMP3 potentiostat.

S4. Spectrophotometric Determination of H_2O_2.

In order to detect and quantify the amount of H_2O_2 formed during controlled potential electrolysis a spectrophotometric method based on the conversion of arylboronic acids into respective phenolates was employed. For the maintaining of an alkaline medium a 150 mM Na_2CO_3 / $NaHCO_3$ buffer solution was prepared and mixed in same volume of a 4 mM *p*-NPBA solution in DMSO, leading the formation of precipitates. Afterwards a known amount of 1 mM H_2O_2 was added to form standard solutions with concentrations ranging from 0.5 µM to 40 µM. The solutions were left to settle and react with H_2O_2 for 30 minutes after addition of H_2O_2. In case of quantification for electrolysis an aliquot (100 µL) were taken from the reaction medium. All solutions were filtered with a 0.45 µm PES filter before spectroscopic detection in order to avoid any interference which may arise from the turbidity in solution. A ThermoFischer Multiskan Go Microplate Spectrophotometer was used throughout the experiments.

S5. Electrochemical Impedance Analysis on TiO_2-NTs/CuTPP-COOH Photoelectrode

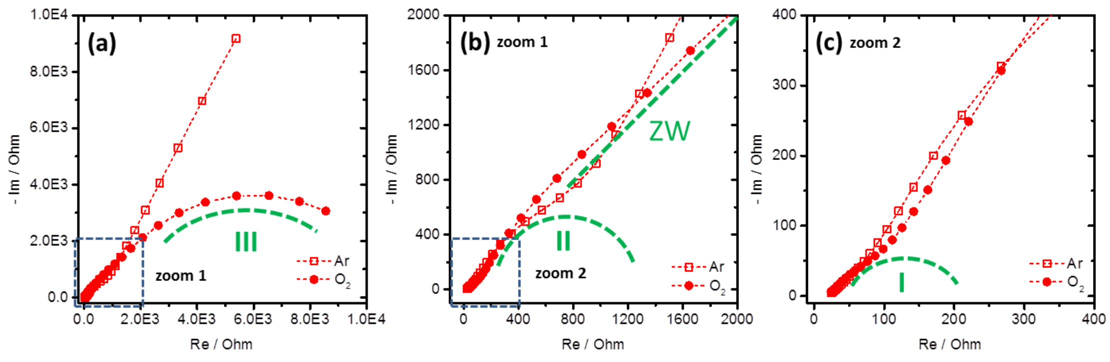

Figure S5. Nyquist plots for illuminated, porphyrin covered TiO_2 NTs at – 0.2 V vs. NHE under Ar (squares) and O_2 (circles) saturation in 0.1 M Na_2SO_4. Symbols represent the experimental data and the dashed lines are only a guide for the eye with no physical meaning. (a) Illustration of the semi-circle (III) at low frequencies between 0.94 Hz and 20 mHz, which is only observable when the electrolyte is saturated with O_2, (b) magnification of the high frequency domain in (a), highlighted with the blue dashed square, to illustrate the second semi-circle (II) at medium frequencies between 65 Hz and 1.4 Hz; The finite length Warburg impedance (ZW) under O_2 (circles) saturation is indicated by a green dashed line with a slope of 45°. (c) Magnification of the high frequency domain in (b), highlighted with the blue dashed square therein, to illustrate the onset of semi-circle (I) at high frequencies between 4.5 kHz and 200 Hz.

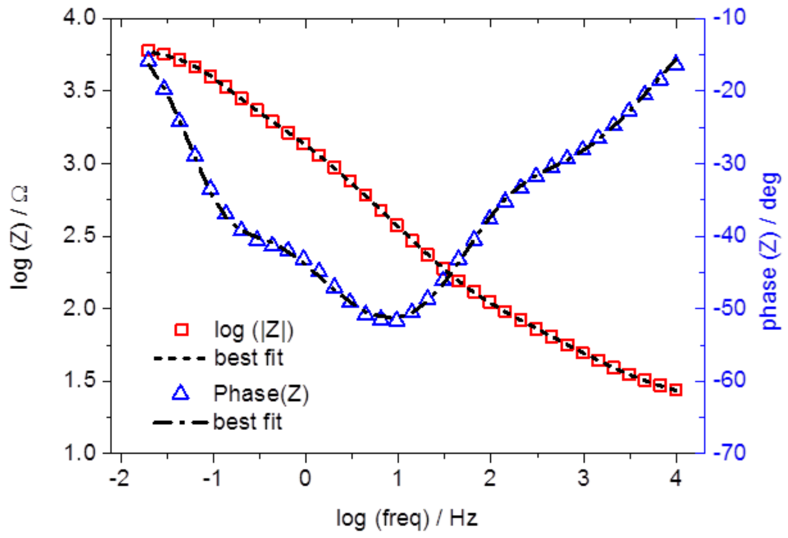

Figure S6. Bode plot at -0.3 V vs. NHE for illuminated, porphyrin covered TiO_2 NTs under O_2 saturation in 0.1 M Na_2SO_4. Symbols represent the experimental data and the dashed lines the corresponding best fit in the frequency range from 10 kHz to 20 mHz.

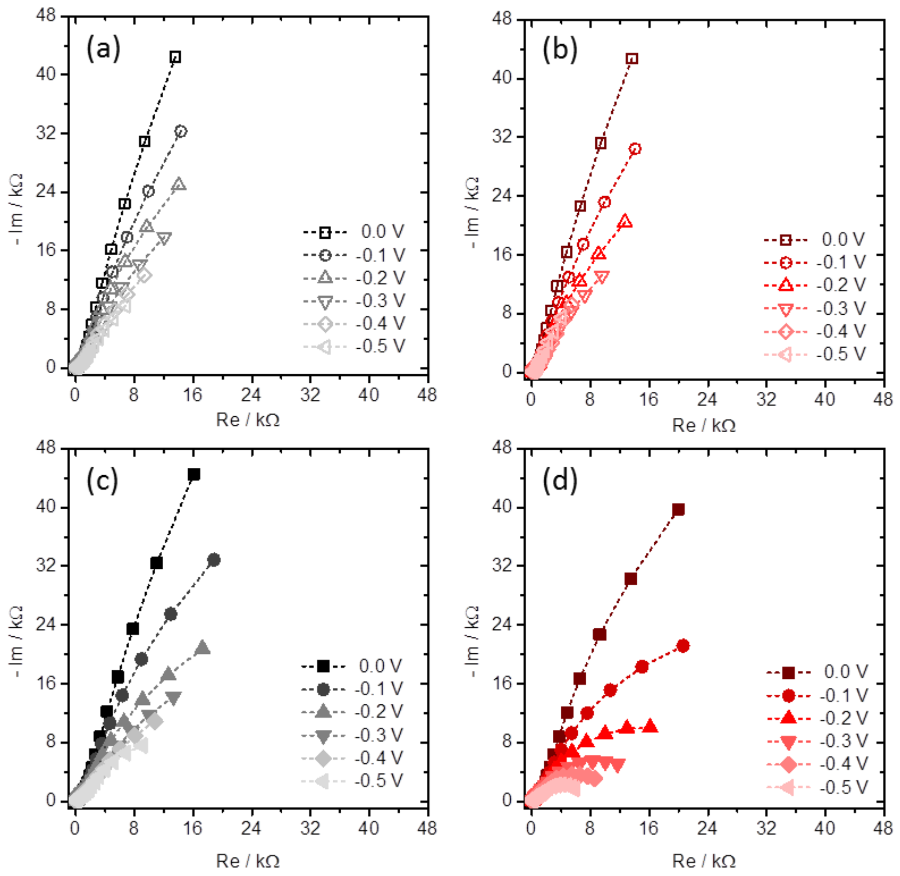

Figure S7. Nyquist plots at different potentials for porphyrin covered TiO_2 NTs in 0.1 M Na_2SO_4: (a) under Ar saturation in the dark, (b) under the Ar saturation and illumination, (c) under the O_2 saturation in dark and (d) under the O_2 saturation and illumination. Measurements were recorded after constant potential for 10 min, ranging from 100 kHz to 20 mHz, with a peak amplitude of ±10 mV.

(Symbols represent the experimental data and the dashed lines are only a guide for the eyes with no physical meaning).

Table S1. Summary of all fitting parameters and their corresponding mean square deviations for the Nyquist plots at different potentials of illuminated, porphyrin covered TiO_2 NTs under O_2 saturation, shown in Figure 4b in the main text. Parameters have been fitted by a *Randomize+Simplex* algorithm with a weighed Z using the EC-Lab Software V11.01.

**Potential**	R_s	R_f	C_f	R_{tr}	C_{nt} [b]	R_r	C_r [b]	ZW	X² / IZI
**/ V [a]**	/ Ω	/ kΩ	/ mF	/ Ω	/ µF	/ kΩ	/ µF	/ Ω.s$^{1/2}$	/ %
**0.2**	19.5	111.6	0.23	2430	96.95	n.d. [c]	n.d.	n.d.	3.30
**0.1**	17.3	25.9	0.31	2217	86.38	n.d.	n.d.	n.d.	0.50
**0.0**	18.6	8.3	0.48	1116	41.56	214.4	24.1	- 22616	0.17
**-0.1**	18.4	3.8	0.69	745	35.73	29.4	13.7	- 2490	0.20
**-0.2**	18.8	4.1	0.68	380	21.28	5.9	17.3	272	0.25
**-0.3**	20.3	3.1	0.69	158	11.14	2.3	26.4	239	0.28

[a] potential vs. NHE. [b] Calculated pseudo-capacitance associated with the CPE. [c] The values could not be determined (n.d.) since at positive potentials the corresponding semi-circle is not developed. The relevancy of the parameter is very low and hence the deviation tolerance very high. This implicates that a great variation of the parameter will not affect the quality of the EEC fit significantly. Hence, the considered parameter is not critical/important or even necessary in the fitting.

References:

[1] C. Allain, D. Schaming, N. Karakostas, M. Erard, J.-P. Gisselbrecht, S. Sorgues, I. Lampre, L. Ruhlmann, B. Hasenknopf, *Dalton Trans.* 2013, 42, 2745−2754.

[2] M. Strohmeier, A. M. Orendt, J. C. Facelli, M. S. Solum, R. J. Pugmire, R. W. Parry, D. M. Grant, *J. Am. Chem. Soc.* 1997, 119, 7114−7120.
